# Short read sequencing assembly revealed the complete mitochondrial genome of *Ischnura elegans* Vander Linden, 1820 (Odonata: Zygoptera)

**DOI:** 10.1080/23802359.2016.1192510

**Published:** 2016-08-21

**Authors:** Wiebke Feindt, Rebecca Herzog, Hans-Jürgen Osigus, Bernd Schierwater, Heike Hadrys

**Affiliations:** a ITZ – Forschungsstätte ‘Alter Bahnhof Schapen’ Braunschweig, University of Veterinary Medicine Hannover, Hannover/Braunschweig, Germany;; b Department of Ecology and Evolutionary Biology, Yale University, New Haven, CT, USA

**Keywords:** A + T rich (control) region, *Ischnura elegans*, iterative mapping, mitochondrial genome, odonata

## Abstract

Damselflies of the genus *Ischnura* emerge as organisms with high potential in ecological, evolutionary and developmental research at the base of flying insects. *Ischnura elegans* and *Ischnura hastata* are for example one of the few odonate species where a complete life cycle over generations can be reared under laboratory conditions. We here report the complete mitochondrial genome of *Ischnura elegans* as a valuable genomic resource for future eco-evo-devo studies at the base of flying insects. The genome has a total length of 15,962 bp and displays all typical features of Odonata (dragonflies and damselflies) mitochondrial genomes in gene content and order as well as A + T content. Start and stop codons of all protein-coding genes are consistent. Most interestingly, we found four intergenic spacer regions and a long A + T rich (control) region of 1196 bp, which is almost double the size of the close relative *Ischnura pumilio*. We assume that the adequate insert size and iterative mapping may be more efficient in assembling this duplicated and repetitive region.

The blue-tailed damselfly *Ischnura elegans* is a small, widely distributed European damselfly of the family Coenagrionidae. The females of this species exhibit a color polymorphism with three different color morphs (e.g. Andrés et al. 2000), which put them in the center of research concerning the evolution of color polymorphism (e.g. Hammers & Van Gossum 2008). Furthermore, the complete life cycle of this species can be cultured in the lab bridging the gaps between developmental, environmental and evolutionary studies (Simon & Hadrys 2013, 2014). It was the first odonate species for which genomic data in terms of ESTs (Simon et al. 2009) and a transcriptome (Chauhan et al. 2014) were available. A complete genome would facilitate state of the art future studies in eco-evo-devo. In a first attempt, we here present the assembly of the mitochondrial genome of *I. elegans*.

Total genomic DNA was extracted from the flight muscles of a single individual using a standard Phenol-Chloroform extraction (Hadrys et al. 1992). The specimen was collected in Schapen, northern Germany (52°16′7.95″N, 10°31′36.37″E). The library preparation and whole genome sequencing was conducted at Yale University in the Center for Genome Analyses (YCGA, http://www.ycga.yale.edu) on an Illumina HiSeq2000 (Illumina Inc.) platform generating 75 bp paired-end reads with an insert size of ∼450 bp. For the assembly of the complete mitochondrial genome Geneious vers. 8.1.5 (http://www.geneious.com) was applied as follows: one published mitochondrial gene sequence served as seed (*cox2*, KC430130), and a fraction of the cleaned reads were mapped onto the seed using iterative mapping. Hereby the iterations were increased with length of the seed sequence (from 5 to 25) as well as the overlap identity, which was initially placed at 90%. A maximum overlap of 45–50 bp was chosen to prevent miss-mapping. In addition, the length of the A + T rich (control) region was confirmed via PCR. The continuous annotation was conducted using the MITOS WebServer (mitos.bioinf.uni-leipzig.de/index.py) and verified via BLAST (Altschul et al. 1990) against GenBank, already published mitochondrial genomes (e.g. Tang et al. 2014; Yu et al. 2014; Chen et al. 2015) and especially against the closest relative *Ischnura pumilio* (NC_021617; Lorenzo-Carballa et al. 2014). Transfer RNAs were predicted using the tRNAscan-SE vers.1.21 Search Server (http://lowelab.ucsc.edu/tRNAscan-SE; Lowe & Eddy 1997) and ARWEN vers. 1.2 (http://mbio-serv2.mbioekol.lu.se/ARWEN; Laslett & Canbäck 2008). Finally, a phylogeny was reconstructed using four other selected Odonata species and *I. elegans* (Figure 1). Based on a concatenated alignment of all 13 protein-coding genes and the rRNA genes a maximum parsimony tree was calculated using PAUP vers. 4.0b10 (Swofford 2002) with a heuristic search under the 50% majority-rule and 1,000 bootstrap replicates. 

**Figure 1.**
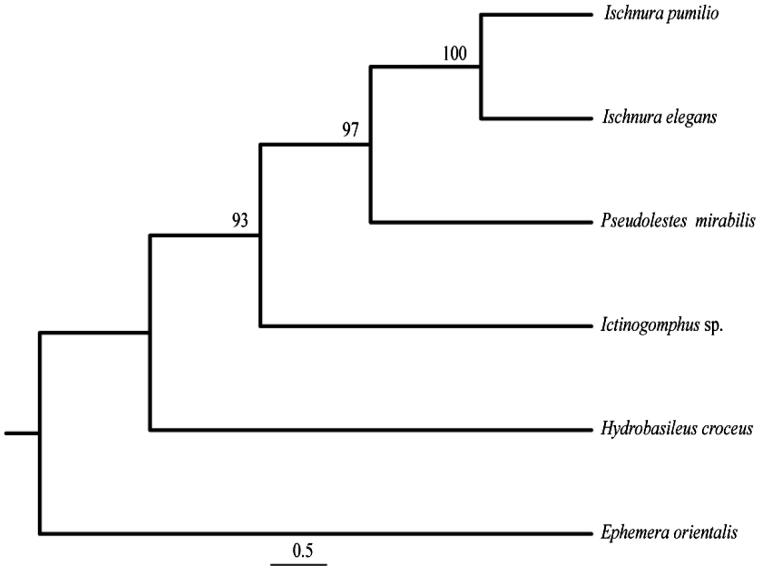
Phylogenetic position of *I. elegans* and *I. pumilio* (NC_021617), *Pseudolestes mirabilis* (NC_020636), *Hydrobasileus croceus* (NC_025758), *Ictinogomphus* sp. (KM244673) using the mayfly *Ephemera orientalis* (NC_012645) as outgroup. MP bootstrap supports are shown for each node. *I. elegans* and *I. pumilio* exhibit 86.4% identity in the mt-genes used.

The present mitogenome has a total length of 15,962 bp (GenBank accession number: KU958378) and is 712 bp longer than *I. pumilio*’s with a high overall similarity of 86.4%. It displays the typical metazoan gene content with 13 protein coding genes (PCGs), 2 rRNA genes (16S and 12S) and 22 tRNA genes with an identical gene order to all other odonate mitogenomes published to date (Table 1). Furthermore, four intergenic spacer regions were detected which are consistent in position with *I. pumilio* and *Megaloprepus caerulatus* (Lorenzo-Carballa et al. 2014; Feindt et al. 2016) but differ in size. Base composition of the *I. elegans* mitogenome is AT biased (A: 40.3%, T: 32.5%, G: 12.3%, C: 14.8%) so as all protein coding genes (71.4%), rRNAs (75.1%) and tRNAs (72.1%) on average. Except for *cox1* (TTA) all PCGs initiate with standard invertebrate mitochondrial start codons: *cox3*, *nad4*, *nad4L* and *cytb* with ATG; *nad2*, *cox2*, *nad5*, with ATT; *atp8*, *atp6*, *nad3*, *nad1* with TTG; and *nad6* with ATC. Complete stop codons terminate nine genes (TAA: *nad2*, *cox1*, *atp8*, *atp6*, *nad4L*, *nad6*, *cytb*, *nad1*; TAG: *nad3*) whereas four proteins use incomplete stop codons with post-transcriptional polyadenylation (*cox2*, *cox3*, *nad5*, *nad4*). Transfer RNAs vary in size from 65 to 72 bp and all of them fold into the characteristic clover-leaf secondary structure. Overlapping gene junctions were observed for 13 genes, the longest overlap between *atp6* and *atp8* is 13 bp.

**Table 1.** Organization of the mitochondrial genome of *Ischnura elegans*.

Name	Strand	Start position	Stop position	Length (bp)	Start codon	Stop codon
*tRNA–Ile*	+	1	67	67	/	/
*tRNA–Gln*	–	65	132	68	/	/
*tRNA–Met*	+	132	200	69	/	/
*NAD2*	+	201	1, 196	996	ATT	TAA
*tRNA–Trp*	+	1195	1263	69	/	/
*tRNA–Cys*	–	1256	1321	66	/	/
*tRNA–Tyr*	–	1322	1389	68	/	/
*s1*	n. a.	1390	1406	17	/	/
*COX1*	+	1407	2967	1561	TTA	TAA
*tRNA–Leu*	+	2963	3028	66	/	/
*COX2*	+	3029	3716	688	ATT	T(aa)
*tRNA–Lys*	+	3717	3788	72	/	/
*tRNA–Asp*	+	3789	3854	66	/	/
*ATP8*	+	3852	4016	165	TTG	TAA
*ATP6*	+	4004	4687	684	TTG	TAA
*COX3*	+	4687	5473	787	ATG	TA(a)
*tRNA–Gly*	+	5474	5538	65	/	/
*NAD3*	+	5539	5892	354	TTG	TAG
*tRNA–Ala*	+	5891	5957	67	/	/
*tRNA–Arg*	+	5956	6021	66	/	/
*tRNA–Asn*	+	6023	6089	67	/	/
*tRNA–Ser*	+	6089	6159	71	/	/
*tRNA–Glu*	+	6161	6227	67	/	/
*tRNA–Phe*	–	6226	6291	66	/	/
*s2*	n. a.	6292	6303	12	/	/
*NAD5*	–	6304	8020	1717	ATT	T(aa)
*tRNA–His*	–	8021	8086	66	/	/
*NAD4L*	–	8087	9429	1343	ATG	TA(a)
*NADU*	–	9423	9716	294	ATG	TAA
*tRNA–Thr*	+	9719	9784	66	/	/
*s3*	n. a.	9785	9794	10	/	/
*tRNA–Pro*	–	9795	9862	68	/	/
*NAD6*	+	9865	10,380	516	ATC	TAA
*CYTB*	+	10,380	11,513	1134	ATG	TAA
*tRNA–Ser*	+	11,514	11,578	65	/	/
*s4*	n. a.	11,579	11,598	20	/	/
*NAD1*	–	11,599	12,549	951	TTG	TAA
*tRNA–Leu*	–	12,551	12,618	68	/	/
*16S*	–	12,619	13,902	1284	/	/
*tRNA–Val*	–	13,903	13,973	71	/	/
*12S*	–	13,974	14,766	793	/	/
*A + T rich (control) region*	n. a.	14,767	15,962	1196	/	/

The table displays the gene order with information about the gene boundaries as well as start and stop codons, whereas incomplete stop codons are displayed as T(aa). All transfer RNAs are named according their corresponding amino acid. The intergenic spacer regions are named continuously from *s1* to *s4*.

The different length of the presented mt genome compared to *I. pumilio* is mainly based on the almost two times longer A + T rich (control) region. Since we proved the length of the A + T rich (control) region via PCR, we assume that the combination of an appropriate insert size and iterative mapping may be more accurate for the assembly of long repetitive and duplicated regions. These usually tend to challenge genome assembly software. The control region comprises a triplicated motive of in total almost 600 bp, which could only be resolved correctly with consideration to the insert size. This could be an important aspect for future library preparation on Illumina platforms.
